# Prognostic Value of Neutrophil Percentage-to-Albumin Ratio in Patients with Oral Cavity Cancer

**DOI:** 10.3390/cancers14194892

**Published:** 2022-10-06

**Authors:** Chien-An Ko, Ku-Hao Fang, Ming-Shao Tsai, Yi-Chan Lee, Chia-Hsuan Lai, Cheng-Ming Hsu, Ethan I. Huang, Geng-He Chang, Yao-Te Tsai

**Affiliations:** 1 Department of Otorhinolaryngology-Head and Neck Surgery, Chang Gung Memorial Hospital, Chiayi 60040, Taiwan; 2 College of Medicine, Chang Gung University, Taoyuan 330036, Taiwan; 3 Department of Otorhinolaryngology-Head and Neck Surgery, Chang Gung Memorial Hospital, Taoyuan 333423, Taiwan; 4 Department of Otorhinolaryngology-Head and Neck Surgery, Chang Gung Memorial Hospital, Keelung 20401, Taiwan; 5 Department of Radiation Oncology, Chang Gung Memorial Hospital, Chiayi 60040, Taiwan

**Keywords:** neutrophil percentage-to-albumin ratio, oral cavity cancer, biomarker, nomogram, prognosis

## Abstract

**Simple Summary:**

Inflammatory response and nutritional status play crucial roles in oral cavity squamous cell carcinoma (OSCC). Increasing evidences suggest the prognostic utility of neutrophil percentage-to-albumin ratio (NPAR) in human malignancies. In this study, we enrolled 368 patients with operated OSCC to investigate the prognostic role of NPAR. Our results demonstrated that patients with a high NPAR (≥16.93) had worse overall survival (OS) and disease-free survival (DFS), and a high NPAR (≥16.93) was an independent risk factor for poor OS and DFS in multivariate analyses. The nomogram integrating independent clinicopathological variables and NPAR provides accurate OS prediction and feasible application to OSCC management. Given its high availability and cost-effectiveness, the NPAR has potential to serve as a promising prognostic biomarker in patients with OSCC after external validation in a larger cohort.

**Abstract:**

This study investigated preoperative neutrophil percentage-to-albumin ratio (NPAR) for predicting oral cavity squamous cell carcinoma (OSCC) survival. We retrospectively analyzed 368 patients who received curative OSCC surgery between 2008 and 2017. Receiver operating characteristic curve analyses were employed to identify the optimal NPAR cutoff (16.93), and the patients were then separated into low-NPAR and high-NPAR groups. Intergroup differences in survival were determined through Kaplan–Meier analysis and log-rank tests. Disease-free survival (DFS) and overall survival (OS) predictors were identified using Cox proportional-hazards models. A nomogram integrating independent prognostic factors was proposed to increase the accuracy of OS prediction. A high NPAR (≥16.93) was associated with worse median OS and DFS than was a low NPAR (both *p* < 0.001); this finding was confirmed through multivariate analyses (hazard ratio (HR) for OS = 2.697, *p* < 0.001; and HR for DFS = 1.671, *p* = 0.008). The nomogram’s favorable predictive ability was confirmed by the calibration plots and concordance index (0.784). The preoperative NPAR is thus a promising prognostic biomarker in patients with OSCC after external validation in a larger cohort. Our nomogram can facilitate clinical use of the NPAR and provides accurate individualized OS predictions.

## 1. Introduction

The most common malignancy of the head and neck is oral cavity cancer; this type of cancer is also globally the sixth most common [1]. In 2020, the global incidence of oral cavity cancer was 377,713 cases, and 177,757 people died from this cancer [2]. The incidence of oral cavity cancer is estimated to increase by up to 40% by 2040, with a corresponding increase in mortality also predicted [2]. Oral cavity cancer is reportedly most common in South and Southeast Asia and some countries in southern Europe [3]. Histologically, oral cavity squamous cell carcinoma (OSCC) is by far the most frequent type of oral cavity cancer and accounts for more than 90% of cases. Given that early diagnosis and timely treatment are crucial in the management of OSCC, effective and convenient biomarkers that may aid early treatment planning should be identified. 

Several clinicopathological factors have been shown to strongly affect OSCC prognosis; these factors include tumor, node, and metastasis stage [4], cancer histologic grading [5], perineural invasion (PNI) [6], depth of invasion (DOI) [7], and extranodal extension (ENE) [8]. Growing evidence indicates that host nutritional and inflammatory status are prognostically valuable for patients with malignancy [9]. In clinical practice, serum albumin concentration has frequently been used as an indicator of malnutrition [10], and higher all-cause mortality was associated with lower serum albumin concentration in OSCC patients [11]. In addition, peripheral neutrophil count and percentage are indicators of systemic inflammation, and tumor-associated neutrophils can promote OSCC progression and metastasis [12]. Therefore, the consideration of inflammatory and nutritional biomarkers in OSCC prognosis is reasonable. Bernard et al. proposed a novel indicator, the neutrophil percentage-to-albumin ratio (NPAR), that combines peripheral neutrophil percentage and serum albumin concentration; they reported its prognostic significance in patients with rectal cancer who underwent neoadjuvant chemoradiotherapy followed by surgery [13]. Several studies have also suggested that the NPAR has potential for predicting survival outcomes in those with gastrointestinal stromal tumors [14], bladder cancer [15], lung cancer [16], and pancreatic cancer [17]. However, data concerning the NPAR’s prognostic utility for patients with OSCC remain scarce. Therefore, we addressed this gap in the literature by investigating the prognostic value of preoperative NPAR for patients undergoing OSCC surgery. 

## 2. Materials and Methods

### 2.1. Design and Study Population

We used a single-institution, retrospective design to analyze 10 years (1 January 2008 to 31 December 2017) of data for 407 consecutive patients with OSCC diagnosed at our hospital’s department of otorhinolaryngology. The data were analyzed from January to June 2021. Patients satisfying the following inclusion criteria were considered: (1) age > 18 years, (2) new pathological OSCC diagnosis during the aforementioned period of investigation, and (3) receipt of curative surgery for OSCC. A patient was excluded from the study if they (1) had a contraindication for surgery or received a diagnosis of unresectable OSCC (*n* = 5), (2) had received neoadjuvant therapy prior to surgery (*n* = 4), (3) had a previous malignancy or synchronous cancer upon receiving their OSCC diagnosis (*n* = 15), (4) had a history of hematological disease or chronic infection (*n* = 4), (5) had symptoms and signs as well as laboratory test results implicating active infection within the 1 month before surgery (*n* = 3), and (6) had missing data of interest (*n* = 8). After 39 patients were excluded, we analyzed the data of 368 patients. 

### 2.2. Data Collection

From the hospital’s electronic records, we obtained clinical data, and medical staff members reviewed these data. We carefully reviewed the patients’ clinicopathological characteristics, such as sex, age upon receiving the OSCC diagnosis, overall pathological stage according to eighth edition of the American Joint Committee on Cancer’s (AJCC’s) Cancer Staging Manual (2018), lymphovascular invasion (LVI) status, PNI status, ENE status, cancer cell differentiation, primary tumor site, closest surgical margin, and DOI. 

To examine the association between patient NPAR and survival outcomes, we used laboratory data that had been obtained within the 2 weeks before surgery. An automatic biochemistry analyzer, the Cobas 8000 (Roche Diagnostics, Hitachi, Rotkreuz, Switzerland), was used to measure preoperative blood biochemistry values, including albumin concentrations. The Sysmex SE-9000 (Sysmex, Kobe, Japan), a hematology analyzer, was used to quantify circulating blood cells, including the proportion of white blood cells constituted by neutrophils. The following formula was employed to calculate the NPAR from the same blood sample: neutrophil percentage (%) × 100/albumin concentration (g/dL) [13]. In addition to peripheral blood cell levels and tumor characteristics, the host-related factors of interest were comorbidities and personal habits. We used the Charlson comorbidity index (CCI) to define and record underlying comorbidities [18]. Consumption of one or more alcoholic drink each week for 6 or more months was considered to indicate alcohol consumption [19], smoking of 10 or more cigarettes each day for at least 1 year was considered to indicate cigarette smoking [20], and chewing more than one betel nut each day for at least 1 year was considered to indicate a betel nut chewing. The patients were then grouped by their engagement in none, one, or at least two of the aforementioned habits. 

### 2.3. Treatment Plan

All patients underwent radical OSCC resection with synchronous neck dissection as needed. The indications and preferable types of postoperative adjuvant therapy, including radiotherapy (RT) and chemoradiotherapy (CRT), were determined by a multidisciplinary tumor board based on our institution’s guidelines for OSCC treatment [21]. Regarding the RT, conventional fractionation of 1.8–2.0 Gy per fraction was delivered with volumetric modulated arc therapy technique. 

### 2.4. Follow-Up and Survival Endpoints

The time from the date of the curative operation to the last follow-up, the study’s end point (31 December 2020), or death was considered the follow-up period. We defined the survival endpoints as follows: (1) overall survival (OS)—the time from surgery to death by any cause, patient censoring, or the final follow-up; (2) disease-free survival (DFS)—the length of time from surgery to disease recurrence, distant metastasis, patient censoring, or the last follow-up. The following criteria were used to confirm distant metastasis or locoregional OSCC recurrence: (1) pathologically confirmed metastatic or recurrent OSCC or (2) suspicious findings of metastatic or recurrent lesions detected through various imaging studies, such as computed tomography (CT), magnetic resonance imaging (MRI), or positron emission tomography (PET)-CT, in lieu of unavailable pathological proof.

### 2.5. Statistical Analysis

SPSS (v. 21.0; SPSS, Chicago, IL, USA) was employed for the statistical analyses performed in this study. After examining data normality through the Kolmogorov–Smirnov test, we present normally and nonnormally distributed continuous variables and categorical variables as means and standard deviations, medians and interquartile ranges (IQRs), and numbers and percentages, respectively. The optimal NPAR cutoff was determined by conducting receiver operating characteristic (ROC) curve analyses and calculating the areas under the ROC curves (AUCs). Patients were then divided into groups based on this cutoff and the survival in the low-NPAR and high-NPAR groups were compared by using log-rank tests. Cox proportional-hazards models were used to estimate the predictive ability of each clinicopathological variable for DFS and OS. The findings are presented in terms of hazard ratios (HRs) with 95% confidence intervals (CIs). In the multivariate analysis, we included all factors with a *p* of <0.1 in the univariate analysis and considered only two-sided *p* values of <0.05 to indicate statistical significance in the final model. 

### 2.6. Nomogram for OS Prediction

To ensure that our predictions of survival in the context of OSCC were accurate, we created a predictive nomogram containing the variables independently associated with OS in the multivariable analysis; this was done by using the rms package in the R language (v. 5.1–0; Vanderbilt University, Nashville, TN, USA) [22]. The concordance index (C-index) was calculated to indicate the accuracy of proposed nomogram in predicting OS. We also assessed the agreement between the nomogram’s predicted outcomes and the actual survival of the patients by drawing calibration plots.

## 3. Results

### 3.1. Baseline Characteristics

Table 1 lists our cohort’s clinicopathological characteristics. Of the 368 participants, 253 (68.8%) were aged <65 years, and 333 (90.5%) were male. The tongue was the most frequent site of the primary tumor (*n* = 142, 38.6%), with the second most frequent site being the buccal mucosa (*n* = 120, 32.6%). According to the AJCC staging system, more than half of the cohort were in stage III–IV disease (*n* = 231, 62.8%). Lymph node metastasis in the neck was confirmed pathologically in 124 (33.7%) patients, and ENE was observed in 70 (19.1%) patients. The planned treatment course was completed in all patients: 185 (50.3%) patients underwent curative surgery alone, 48 (13.0%) patients also underwent adjuvant RT, and 135 (36.7%) patients also received adjuvant CRT. 

### 3.2. Association between Clinicopathological Characteristics and NPAR

**Figure 1:**
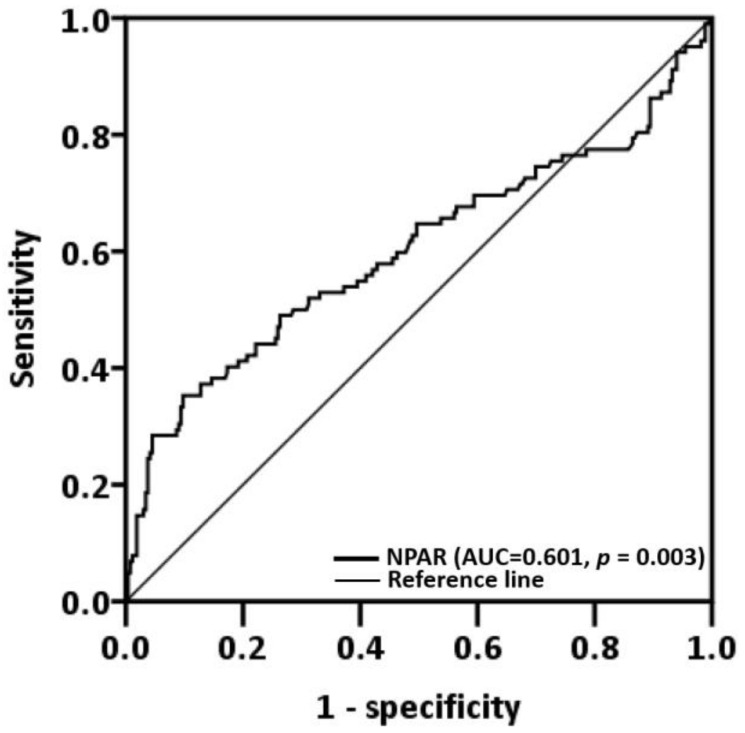
Neutrophil percentage-to-albumin ratio cutoff obtained through receiver operating characteristic curve analysis. Abbreviation: AUC, area under the curve.

The median NPAR was 11.15 (IQR: 8.22–14.49), and, by using ROC analysis with Youden’s J-point for the balance between specificity and sensitivity, we discovered that the optimal cutoff was 16.93 (*p* = 0.003, Figure 1). A low-NPAR (<16.93) group (*n* = 306, 83.2%) and a high-NPAR (≥16.93) group (*n* = 62, 16.8%) were created on the basis of the aforementioned cutoff (Table 1). Patients with a high NPAR were more prone to have stage III or IV disease (*p* = 0.001), late T classification (*p* < 0.001), late N classification (*p* = 0.007), PNI (*p* = 0.001), ENE (*p* < 0.001), LVI (*p* < 0.001), poor cell differentiation (*p* = 0.024), DOI ≥ 10 mm (*p* < 0.001), tongue and buccal cancer (*p* = 0.016), and the need for adjuvant therapy (*p* < 0.001). 

### 3.3. Significance of NPAR for OS

**Figure 2:**
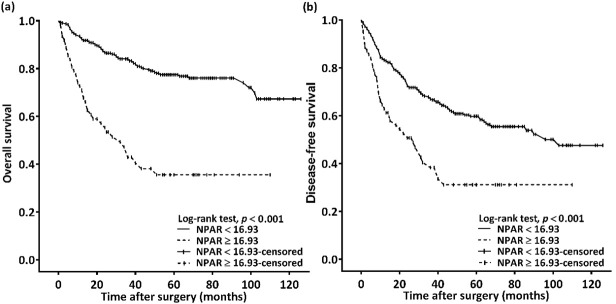
(**a**) Overall survival and (**b**) disease-free survival Kaplan–Meier curves for patients with oral squamous cell carcinoma classified on the basis of preoperative NPAR. Prognosis was significantly poorer in patients with an NPAR of ≥16.93. Abbreviation: NPAR, neutrophil percentage-to-albumin ratio.

**Figure 3:**
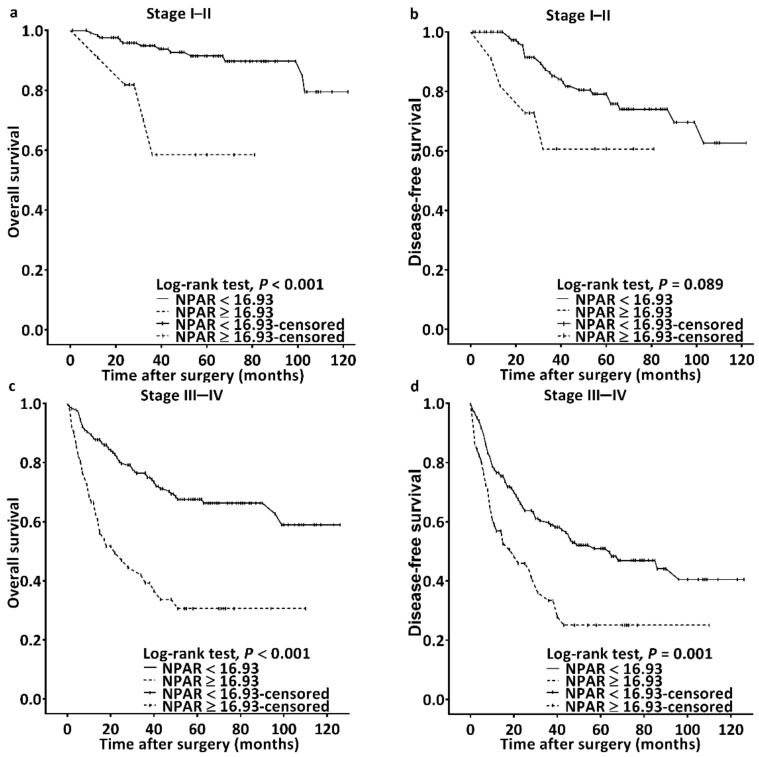
Kaplan–Meier survival curves for patients classified based on cutoff of NPAR and cancer stage. Superior overall survival was discovered in the low-NPAR group among patients with (**a**) stages I–II and (**c**) stages III–IV OSCC (both *p* < 0.001); a similar finding was obtained for disease-free survival for (**b**) stages I–II and (**d**) stages III–IV OSCC (*p* = 0.089 and 0.001, respectively). Abbreviation: NPAR, neutrophil percentage-to-albumin ratio.

We observed a median (IQR) follow-up duration of 44 (22–71) months; during the follow-up, 102 (27.7%) patients passed away. Median OS was estimated using Kaplan–Meier analysis to be 32 (95% CI: 19–45) months for patients with an NPAR of ≥16.93 and >103 months for those with an NPAR of <16.93; the log-rank test identified this as a significant difference in OS (*p* < 0.001, Figure 2a). Table 2 details the OS–clinicopathological variable correlations. Stage IV disease, PNI, LVI, poor cell differentiation, the need for CRT, CCI ≥ 2, and an NPAR of ≥16.93 were discovered through univariate analysis to be significantly correlated with poor OS. In the multivariable analysis, stage IV disease (HR: 4.913; 95% CI: 2.091–11.541; *p* < 0.001), PNI (HR: 1.707; 95% CI: 1.099–2.650; *p* = 0.017), poor cell differentiation (HR: 2.332; 95% CI: 1.372–3.964; *p* = 0.002), CCI ≥ 2 (HR: 2.239; 95% CI: 1.327–3.778; *p* = 0.003), and NPAR ≥ 16.93 (HR: 2.697; 95% CI: 1.761–4.130; *p* < 0.001) were identified as significant independent risk factors for poor OS. To investigate the effect of the cancer stage–NPAR interaction on OS, we created Kaplan–Meier survival curves after stratification by cancer stage and NPAR (Figure 3). OS was superior in the low-NPAR group both for stages I–II (Figure 3a) and for stages III–IV OSCC (Figure 3c; both *p* < 0.001) 

### 3.4. Significance of NPAR for DFS

The Kaplan–Meier survival curve analysis showed that the estimated median DFS was 103 months in the patients with an NPAR of <16.93 and 27 (95% CI 15–39) months in those with an NPAR of ≥16.93 (*p* < 0.001, Figure 2b). Table 3 presents the associations between clinicopathological variables and DFS. DFS was discovered to be significantly associated with stage IV disease, PNI, LVI, poor cell differentiation, the need for CRT, and an NPAR of ≥16.93 in the univariate analysis. Poor DFS was independently predicted by stage IV disease (HR: 2.581; 95% CI: 1.504–4.428; *p* = 0.001), poor cell differentiation (HR: 2.030; 95% CI: 1.308–3.149; *p* = 0.002), and an NPAR of ≥16.93 (HR: 1.671; 95% CI: 1.142–2.444; *p* = 0.008) in the multivariate analysis. When the DFS curves were stratified by cancer stage and NPAR, we discovered that the patients in the low-NPAR group had better DFS than patients in the high-NPAR group (Figure 3b,d: stages I–II and stages III–IV; *p* = 0.089 and 0.001, respectively).

### 3.5. Establishment of NPAR-Based Nomogram

**Figure 4:**
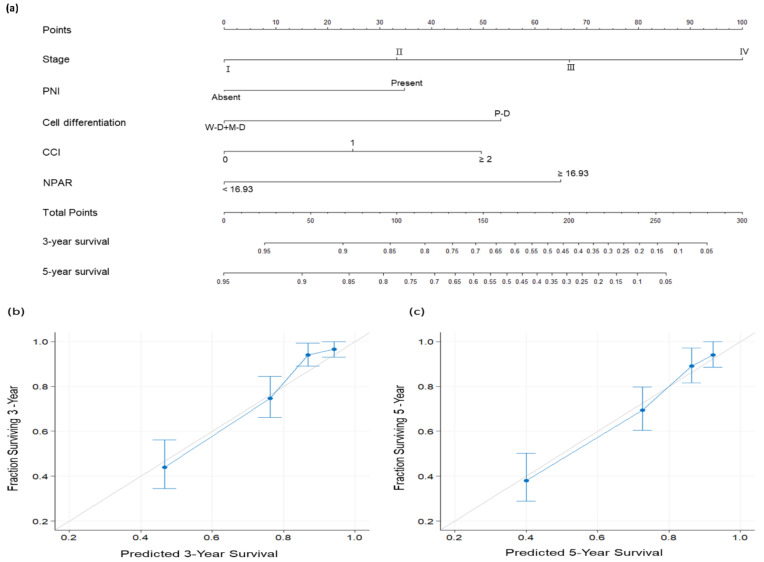
(**a**) Nomogram predicting overall survival (OS) based on neutrophil percentage-to-albumin ratio and prognostic factors identified in multivariate analysis. The degree of risk contributed by each variable is indicated by the line segment and its uppermost points. The total number of points is the sum of each variable’s points. Drawing a vertical line from the calculated total number of points gives the likelihood of 3-year and 5-year OS. Calibration plots for (**b**) 3-year and (**c**) 5-year OS. The gray line at 45° reflects perfectly accurate prediction; the predictive outcomes of the nomogram are depicted by the blue line. The performance of the nomogram and the 95% confidence intervals for the OS predictions are shown as blue dots with bars, respectively. Abbreviations: CCI, Charlson comorbidity index; M–D, moderately differentiated; NPAR, neutrophil percentage-to-albumin ratio; P–D, poorly differentiated; PNI, perineural invasion; W–D, well differentiated.

To provide physicians with a quantitative method for predicting three-year and five-year OS for a given patient, a nomogram incorporating the ostensibly significant factors identified in the multivariate analysis—overall stage, PNI, cancer cell differentiation, CCI, and NPAR—was established (Figure 4). For comparison, we also calculated the C-index of an AJCC staging-system-based nomogram; the C-index of this nomogram and that of our NPAR-based nomogram were 0.693 (95% CI: 0.665–0.721) and 0.784 (95% CI: 0.754–0.814), respectively. The AUC of the NPAR-based nomogram model was 0.793 (cutoff: 122; sensitivity: 77.5%; specificity: 69.8%). The calibration plots thus indicated favorable agreement between the nomogram’s three-year and five-year OS predictions (Figure 4b,c, respectively) and the actual survival outcomes. All these findings suggest that the NPAR-based nomogram has high discrimination ability and can provide accurate individualized OS prediction for patients undergoing OSCC surgery. 

## 4. Discussion

According to our literature review, our study is the first to evaluate the preoperative prognostic value of the NPAR in OSCC. Using clinical data derived from a large cohort of patients with OSCC treated with surgery, we obtained several findings. A preoperative NPAR of ≥16.93 was associated with adverse clinicopathological factors—later cancer stage, PNI, ENE, LVI, poor cell differentiation, late T and N classification, and a DOI of ≥10 mm—suggesting that preoperative systemic inflammation and nutrition are strong indicators of OSCC aggressiveness. According to the multivariate analysis, poor OS and DFS are associated with a high preoperative NPAR (≥16.93; HR = 2.697 and 1.671, respectively). The NPAR retained its predictive ability, even for cancer-stage subgroups. Finally, as confirmed by C-index values and calibration plots, our novel NPAR-based nomogram provides accurate three-year and five-year OS predictions and facilitates clinical application of the NPAR in OSCC management. Because the NPAR can be easily obtained from routine peripheral blood tests conducted before surgery, it has potential to serve as a simple and cost-effective biomarker in clinical practice. Our study results may help clinicians predict the outcomes of conventional curative treatment and conduct more personalized management for patients with high risks, particularly in those with early-stage OSCC. 

Researchers have investigated the ability of the NPAR to predict survival outcomes in individuals with cancer, and they have obtained evidence that supports our study’s findings. In a retrospective study of 145 individuals with pancreatic adenocarcinoma undergoing palliative treatment, the NPAR was an independent predictor of survival, whereas the platelet–lymphocyte and neutrophil–lymphocyte ratios were not [17]. Similarly, scholars have demonstrated an association between poor survival outcomes and an elevated NPAR in a group of individuals with bladder cancer receiving neoadjuvant chemotherapy and cystectomy [15] as well as in a group of patients receiving surgery for stage I–III colorectal adenocarcinoma [23]. In addition, in a study of 98 patients with rectal cancer receiving neoadjuvant CRT or RT alone, the NPAR independently predicted complete remission [13]. The NPAR was found to be more prognostically sensitive than serum albumin or neutrophil levels alone [24]. Because various factors such as chronic liver disease and changes in body fluid volume can influence serum neutrophil and albumin levels, the NPAR, which combines neutrophil percentage and serum albumin concentration, may have lower measurement variability and be more reliable than serum albumin or neutrophils alone. Furthermore, studies have indicated that immunonutrition supplements and vigorous nutritional supports before and during anticancer treatments may provide survival benefits in terms of functional capacity and tumor recurrence for patients with head and neck cancer [25,26]. Thus, active nutritional interventions should be considered in the management of OSCC patients with a high NPAR, and whether NPAR could be an immunonutritional marker for rapid identifying patients who will benefit from nutritional interventions warrants further investigation. In future prospective clinical trials of patients with OSCC, the role of the preoperative NPAR or changes therein for predicting the outcomes of targeted therapy or immunotherapy should also be evaluated. 

Although studies have indicated the prognostic significance of the NPAR in various diseases, the mechanisms underlying the association between the NPAR and OSCC prognosis are unknown. Several studies have discovered significant associations between systemic inflammatory responses and carcinogenesis [27,28], and high neutrophil counts promote cancer progression and metastasis through several pathways, such as stimulation of angiogenesis and impairment of T-cell-dependent antitumor immunity [29,30]. In their study involving 309 patients with OSCC who underwent surgery, Diao et al. identified significant associations between a high neutrophil count and poor OS and DFS [31]. In addition to immunoinflammation, nutritional status is correlated with cancer survival. Regarding head and neck cancer, more than 60% of patients have malnutrition when they receive their diagnosis [32], which may be explained by cancer-related oral dysfunction, excessive alcohol consumption, or cachexia [33]. Malnutrition is partially reflected by a lower albumin concentration and can impair the antitumor immune response, increase susceptibility to infection and postoperative complications [34,35], and influence the intensity of treatment that a patient can tolerate [36]. Lim et al. reported that low pretreatment albumin levels were associated with poor OS and DFS in a group of 338 patients treated for head and neck cancer [37]. A high NPAR can reflect an elevated circulating neutrophil count, a low albumin concentration, or both, which are correlated with poorer OSCC survival outcomes. In addition, we discovered significant associations between a high NPAR of ≥16.93 and adverse pathological characteristics, such as late T and N classification, and these findings may help researchers discover why the NPAR can predict survival outcomes in OSCC. That is to say, greater tumor burden may be accompanied with stronger systemic inflammatory response [38,39], recruitment of neutrophils [40], increased catabolism, and depletion of serum albumin, which may contribute to the association between a high NPAR and poor OSCC survival; however, the definite mechanism warrants further investigation.

The most commonly used system for estimating survival in OSCC is currently the AJCC staging system [41], which focuses on the characteristics of a tumor without considering other factors influencing prognosis, for example, nutritional status and inflammatory response [11,27]. Therefore, despite the AJCC staging system’s ability to estimate survival outcomes, prognostic heterogeneity exists within the same staging groups [42], suggesting that survival estimation may be improved through the consideration of host factors. The nomogram model, in which various clinicopathological and demographic information is integrated, has emerged as a simple and reliable predictive tool for clinicians aiming to personalize oncological treatment [43]. After identifying relevant factors through multivariate analysis, we created a nomogram integrating the NPAR and independent predictive factors; this nomogram favorably predicted three-year and five-year OSCC OS. We also conducted a series of assessments with the constructed model. The nomogram’s AUC and C-index are 0.793 and 0.784, respectively, suggesting its high performance. To confirm the degree of calibration of the model, we drew calibration plots and the results revealed the high consistency between the actual survival outcomes and the model-predicted OS probabilities. In addition, the efficacy of the NPAR in our nomogram highlights the informative roles of inflammatory and nutritional factors in OSCC prognosis. In summary, the proposed nomogram integrating the NPAR and clinicopathological variables may facilitate clinical application of the NPAR and provide accurate individualized OS predictions for patients with OSCC, leading to personalized treatment. 

Several study limitations should be mentioned. First, bias may have been introduced by the retrospective single-institute design. Second, the results have not been validated with an independent patient cohort. The study results could be externally validated using an independent data set to add more evidence of the prognostic role of the NPAR in OSCC. Third, various NPAR cutoffs are reported in the literature, and the lack of consensus on the optimal cutoff for predicting survival in OSCC has impaired the general clinical applicability of the NPAR. Finally, although we included personal habits in the survival analysis, the quantitative levels of alcohol and tobacco consumptions together with their status of use (current vs. former use) were not available in our database. Further studies with more quantitative measurements of the aforementioned personal habits are necessary to explore the dosage effect of personal habits on the prognosis of OSCC. Before clinical use of the NPAR can be recommended, our findings should be validated through large-scale prospective randomized controlled studies. 

## 5. Conclusions

Our results indicate that preoperative NPAR is a promising prognostic biomarker for OSCC surgery, highlighting that inflammatory and nutritional status should be considered in OSCC prognosis. The NPAR-based nomogram established in this study provides accurate individualized OS prediction and facilitates convenient clinical application of the ratio. One major advantage of the NPAR is that it is simple and inexpensive to measure; it should thus be considered a feasible biomarker in oncologic research and personalized treatment planning for OSCC. Before our study results can be applied to clinical practice, researchers should validate them through large prospective multicenter studies.

## Figures and Tables

**Table 1:** Baseline characteristics of study participants and the stratification based on NPAR cutoff value.

Variable	Total	Number of Patients		*p* Value
		NPAR < 16.93 *n* = 306	NPAR ≥ 16.93 *n* = 62	
Sex				0.670 ^a^
Men	333 (90.5%)	276 (90.2%)	57 (91.9%)	
Women	35 (9.5%)	30 (9.8%)	5 (8.1%)	
Age				0.189 ^a^
<65	253 (68.8%)	206 (67.3%)	47 (75.8%)	
≥65	115 (31.2%)	100 (32.7%)	15 (24.2%)	
AJCC stage				0.001 ^a^
I–II	137 (37.2%)	126 (41.2%)	11 (17.7%)	
III–IV	231 (62.8%)	180 (58.8%)	51 (82.3%)	
T classification				<0.001 ^a^
T1–T2	173 (47.1%)	159 (52.0%)	14 (22.6%)	
T3–T4	195 (52.9)	147 (48.0%)	48 (77.4%)	
N classification				0.007 ^a^
N0	244 (66.3%)	212 (69.3%)	32 (51.6%)	
N1–N3	124 (33.7%)	94 (30.7%)	30 (48.4%)	
Presence of PNI				0.001 ^a^
No	278 (75.5%)	241 (78.8%)	37 (59.7%)	
Yes	90 (24.5%)	65 (21.2%)	25 (40.3%)	
Presence of ENE				<0.001 ^a^
No	297 (80.9%)	257 (84.0%)	40 (64.5%)	
Yes	70 (19.1%)	48 (16.0%)	22 (35.5%)	
Presence of LVI				<0.001 ^a^
No	345 (93.7%)	294 (96.1%)	51 (82.3%)	
Yes	23 (6.3%)	12 (3.9%)	11 (17.7%)	
Cancer histologic grading				0.024 ^a^
W–D/M–D	327 (88.9%)	277 (90.5%)	50 (80.6%)	
P–D	41 (11.1%)	29 (9.5%)	12 (19.4%)	
Closest margin				0.466 ^a^
≥5 mm	269 (73.1%)	226 (73.9%)	43 (69.4%)	
<5 mm	99 (26.9%)	80 (26.1%)	19 (30.6%)	
DOI ≥ 10 mm				<0.001 ^a^
No	198 (53.8%)	185 (60.5%)	13 (21.0%)	
Yes	170 (46.2%)	121 (39.5%)	49 (79.0%)	
Tumor subsites				0.016 ^a^
Tongue	142 (38.6%)	116 (37.9%)	26 (41.9%)	
Buccal mucosa	120 (32.6%)	93 (30.4%)	27 (43.5%)	
Other	106 (28.8%)	97 (31.7%)	9 (14.5%)	
Personal habits				0.648 ^a^
No exposure	44 (11.9%)	38 (12.4%)	6 (9.7%)	
One exposure	22 (5.9%)	17 (5.6%)	5 (8.1%)	
Two or all exposure	302 (82.2%)	251 (82.0%)	51 (82.3%)	
Treatment modality				<0.001 ^a^
Surgery only	185 (50.3%)	169 (55.2%)	16 (25.8%)	
Surgery then RT	48 (13.0%)	43 (14.1%)	5 (8.1%)	
Surgery then CRT	135 (36.7%)	94 (30.7%)	41 (66.1%)	
CCI				0.849 ^a^
0	198 (53.8%)	163 (53.3%)	35 (56.5%)	
1	112 (30.4%)	95 (31.0%)	17 (27.4%)	
≥2	58 (15.8%)	48 (15.7%)	10 (16.1%)	
Albumin (g/dL), median (IQR)	4.48 (4.19–4.69)	4.50 (4.27–4.70)	4.13 (3.66–4.42)	<0.001 ^b^
WBC (×10^3^/μL), median (IQR)	7.80 (6.20–9.70)	7.20 (6.00–8.70)	11.50 (10.40–12.95)	<0.001 ^b^
Neutrophil (×10^3^/μL), median (IQR)	4.81 (3.59–6.34)	4.38 (3.44–5.55)	8.51 (7.70–9.83)	<0.001 ^b^
Lymphocyte (×10^3^/μL), median (IQR)	2.05 (1.62–2.61)	2.09 (1.66–2.63)	1.97 (1.49–2.54)	0.125 ^b^
Survival in months, median (IQR)	11.15 (8.22–14.49)	49.00 (26.75–75.00)	23.00 (10.00–48.75)	<0.001 ^b^

Abbreviations: AJCC, American Joint Committee on Cancer; CCI, Charlson comorbidity index; CRT, chemoradiotherapy; DOI, depth of invasion; ENE, extranodal extension; IQR, interquartile range; LVI, lymphovascular invasion; M–D, moderately differentiated; NPAR, neutrophil percentage-to-albumin ratio; P–D, poorly differentiated; PNI, perineural invasion; RT, radiotherapy; W–D, well differentiated; WBC, white blood cell count. ^a^ the Chi-square test. ^b^ the Mann–Whitney U test (Z-test: Albumin: −5.589; WBC: −10.813; Neutrophil: −11.656; Lymphocyte: −1.534; Survival in months: −5.211).

**Table 2:** Univariate and multivariate analysis of OS of 368 patients with oral cavity squamous cell carcinoma.

Variables	5-Year OS	Univariate Analysis		Multivariate Analysis	
		HR (95% CI)	*p*	HR (95% CI)	*p*
Sex					
Women	73.4%	Reference		Reference	
Men	70.1%	1.165 (0.587–2.312)	0.662	0.720 (0.353–1.468)	0.366
Age (years)					
<65	70.0%	Reference		Reference	
≥65	71.3%	1.028 (0.679–1.556)	0.898	1.047 (0.669–1.638)	0.840
AJCC stage					
I	91.7%	Reference		Reference	
II	84.2%	1.584 (0.595–4.222)	0.358	2.057 (0.760–5.568)	0.156
III	82.9%	1.798 (0.693–4.664)	0.228	1.958 (0.729–5.258)	0.183
IV	52.1%	6.316 (3.039–13.125)	<0.001	4.913 (2.091–11.541)	<0.001
Presence of PNI					
No	76.7%	Reference		Reference	
Yes	51.1%	2.669 (1.794–3.971)	<0.001	1.707 (1.099–2.650)	0.017
Presence of LVI					
No	72.8%	Reference		Reference	
Yes	25.4%	3.692 (2.050–6.649)	<0.001	1.713 (0.907–3.235)	0.097
Cancer histologic grading					
W–D/M–D	74.0%	Reference		Reference	
P–D	43.0%	2.992 (1.861–4.810)	<0.001	2.332 (1.372–3.964)	0.002
Treatment modality					
Surgery only	82.6%	Reference		Reference	
Surgery then RT	76.1%	1.609 (0.806–3.214)	0.178	0.806 (0.379–1.715)	0.576
Surgery then CRT	51.6%	3.838 (2.473–5.957)	<0.001	1.089 (0.587–2.019)	0.787
Tumor location					
Tongue	72.3%	Reference			
Buccal mucosa	69.3%	1.137 (0.714–1.811)	0.590		
Other sites	69.6%	1.102 (0.682–1.782)	0.692		
Closest margin					
≥5 mm	72.5%	Reference			
<5 mm	65.1%	1.377 (0.911–2.081)	0.129		
Personal habits					
No exposure	71.0%	Reference			
One exposure	55.9%	1.746 (0.723–4.214)	0.215		
Two or more exposure	71.5%	1.101 (0.587–2.068)	0.764		
CCI					
0	74.1%	Reference		Reference	
1	71.1%	1.227 (0.775–1.941)	0.383	1.262 (0.773–2.060)	0.352
≥2	58.5%	1.905 (1.170–3.100)	0.010	2.239 (1.327–3.778)	0.003
NPAR					
<16.93	77.5%	Reference		Reference	
≥16.93	35.6%	4.063 (2.693–6.129)	<0.001	2.697 (1.761–4.130)	<0.001

Abbreviations: AJCC, American Joint Committee on Cancer; CCI, Charlson comorbidity index; CI, confidence interval; CRT, chemoradiotherapy; HR, hazard ratio; LVI, lymphovascular invasion; M–D, moderately differentiated; NPAR, neutrophil percentage-to-albumin ratio; OS, overall survival; P–D, poorly differentiated; PNI, perineural invasion; RT, radiotherapy; W–D, well differentiated.

**Table 3:** Univariate and multivariate analysis of DFS of 368 patients with oral cavity squamous cell carcinoma.

Variables	5-Year DFS	Univariate Analysis		Multivariate Analysis	
		HR (95% CI)	*p*	HR (95% CI)	*p*
Sex					
Women	65.2%	Reference		Reference	
Men	53.7%	1.196 (0.690–2.074)	0.523	0.940 (0.533–1.659)	0.831
Age (years)					
<65	52.8%	Reference		Reference	
≥65	59.5%	0.810 (0.574–1.144)	0.233	0.869 (0.611–1.236)	0.434
AJCC stage					
I	69.1%	Reference		Reference	
II	74.0%	0.700 (0.361–1.357)	0.291	0.778 (0.399–1.515)	0.460
III	63.6%	1.114 (0.618–2.005)	0.720	1.323 (0.716–2.442)	0.372
IV	39.4%	2.396 (1.564–3.670)	<0.001	2.581 (1.504–4.428)	0.001
Presence of PNI					
No	58.4%	Reference		Reference	
Yes	43.8%	1.548 (1.098–2.182)	0.013	1.095 (0.750–1.600)	0.638
Presence of LVI					
No	56.2%	Reference		Reference	
Yes	25.4%	1.976 (1.118–3.495)	0.019	1.372 (0.753–2.499)	0.302
Cancer histologic grading					
W–D/M–D	57.8%	Reference		Reference	
P–D	33.5%	2.288 (1.508–3.470)	<0.001	2.030 (1.308–3.149)	0.002
Treatment modality					
Surgery only	63.1%	Reference		Reference	
Surgery then RT	60.7%	1.144 (0.679–1.927)	0.614	0.646 (0.367–1.137)	0.130
Surgery then CRT	41.5%	2.035 (1.460–2.835)	<0.001	1.293 (0.743–1.276)	0.340
Tumor location					
Tongue	60.4%	Reference			
Buccal mucosa	51.8%	1.153 (0.789–1.686)	0.461		
Other sites	50.5%	1.320 (0.904–1.929)	0.151		
Closest margin					
≥5 mm	57.4%	Reference			
<5 mm	48.4%	1.287 (0.922–1.797)	0.138		
Personal habits					
No exposure	7.5%	Reference			
One exposure	46.4%	1.735 (0.787–3.822)	0.172		
Two or more exposure	53.8%	1.540 (0.887–2.673)	0.125		
CCI					
0	53.7%	Reference			
1	60.1%	0.849 (0.586–1.230)	0.386		
≥2	50.0%	1.177 (0.777–1.782)	0.442		
NPAR					
<16.93	59.7%	Reference		Reference	
≥16.93	31.1%	2.215 (1.540–3.186)	<0.001	1.671 (1.142–2.444)	0.008

Abbreviations: AJCC, American Joint Committee on Cancer; CCI, Charlson comorbidity index; CI, confidence interval; CRT, chemoradiotherapy; DFS, disease-free survival; HR, hazard ratio; LVI, lymphovascular invasion; M–D, moderately differentiated; NPAR, neutrophil percentage-to-albumin ratio; P–D, poorly differentiated; PNI, perineural invasion; RT, radiotherapy; W–D, well differentiated.

## Data Availability

The data presented in this study are available on request from the corresponding author.
